# Myasthenia Gravis Associated with Acute Hepatitis E Infection in Immunocompetent Woman

**DOI:** 10.3201/eid2005.131551

**Published:** 2014-05

**Authors:** Aude Belbezier, Alban Deroux, Françoise Sarrot-Reynauld, Sylvie Larrat, Laurence Bouillet

**Affiliations:** Centre Hospitalier Universitaire de Grenoble, Grenoble, France (A. Belbezier, A. Deroux, F. Sarrot-Reynauld, S. Larrat, L. Bouillet);; Université Joseph Fourier Grenoble 1, Grenoble (F. Sarrot-Reynauld, S. Larrat, L. Bouillet)

**Keywords:** hepatitis E virus, myasthenia gravis, MuSK protein, antibodies, immunoglobulin, intravenous, ribavirin, France, immunocompetent, HEV, hepatitis, viruses, IVIG

**To the Editor:** Hepatitis E virus (HEV) is a common cause of acute hepatitis in developing countries. The course of acute hepatitis E is usually benign, except in pregnant women and in immunocompromised patients, who are prone to a lethal or chronic outcome of the disease. Since 2001, hepatitis E has been emerging in industrialized countries, and neurologic manifestations such as Guillain-Barré syndrome, brachial neuritis, transverse myelitis, and cranial nerve palsies have been reported in patients with acute or chronic forms of the disease (*1**–**6*). Most cases with neurologic manifestations have been characterized by infection with genotype 3 HEV. Data are not available to indicate whether this association between HEV infection and neurologic manifestations is related to a specific antigenic stimulus provided by HEV or is linked to the more comprehensive assessment for such neurologic conditions that is available in industrialized countries or to a reporting bias. We report a case of HEV infection in an immunocompetent woman who had muscle-specific kinase (MuSK) antibody–positive myasthenia gravis associated with HEV replication.

A 33-year-old woman was hospitalized in France for subacute asthenia and intermittent symptoms including dysarthria, dysphagia, muscle weakness, and diplopia. She had no family history of autoimmune disease and no notable personal medical history; she had not received any recent vaccinations and had not traveled outside France during the previous year. Physical examination showed no pyramidal, vestibular, or cerebellar syndromes, and all tendon reflexes were typical.

On admission, the patient’s liver function tests showed elevated alanine transaminase (190 UI/L). Test results were within reference ranges for aspartate aminotransferase, alkaline phosphatase, gamma-glutamyl transpeptidase, and creatine kinase levels. Antibody tests were negative for hepatitis B virus, hepatitis C virus, HIV, human T-lymphotropic viruses 1 and 2, and *Treponema pallidum*, but testing for cytomegalovirus and Epstein-Barr virus showed previous exposure. Serum samples were positive for HEV IgM (index 11) and negative for HEV IgG (index 0.71) by Adaltis microplate ELISA (EIAgen; Adaltis, Casalecchio Di Reno, Italy). HEV RNA was detected in serum and fecal samples by using HEV reverse transcription PCR (RT-PCR) (Ceeram, La Chapelle sur Erdre, France) on the SmartCycler II instrument (Cepheid, Sunnyvale, CA, USA). Sequencing studies showed that the HEV strain belonged to genotype 3f.

Brain magnetic resonance imaging results were normal, and results for analysis of cerebrospinal fluid were normal (leukocytes <5 cells/µL, glucose 3.2 mmol/L, protein 38 mg/dL, HEV RT-PCR negative). A nerve conduction study, performed even though the patient was not symptomatic, did not find any abnormality. Pharmacologic testing with prostigmine (0.5 mg intravenously) did not result in symptom improvement. Computed tomography scan of the mediastinum showed no thymoma. Test results for anti-acetylcholine receptor antibodies were negative; however, results were positive for MuSK antibodies (18.7 U/mL by radioimmunoassay; RSR Ltd., Cardiff, Wales, UK). These data, combined with physical examination, confirmed the diagnosis of myasthenia gravis.

Given this association of myasthenia and acute HEV infection, we suspected the potent role of HEV infection in the neurologic symptoms. Therapy was started with ribavirin (1,000 mg/day for 1 month) and intravenous immunoglobulin (IVIG) (1 g/kg/d for 2 days). Alanine transaminase levels rapidly decreased, and after 3 months, test results for HEV IgM and HEV RT-PCR in serum were negative. IVIG treatment relieved symptoms in the short term, but symptoms returned 15 days after treatment ended, requiring continuation of IVIG every 4 weeks for 6 months. Four months after the last infusion of IVIG, the patient reported mild 4-limb fatigability after exercise, but results of objective neurologic examination were normal. Results for MuSK antibodies were still positive, and treatment with azathioprine (150 mg/d) was started. Three weeks later, the patient required another infusion of IVIG for difficulty in swallowing, dyspnea, and 4-limb weakness, but she was free of symptoms for the remaining 5 months of follow-up.

In conclusion, we describe a case of anti-MuSK myasthenia gravis associated with acute HEV in a young, immunocompetent patient in France. Because myasthenia gravis with MuSK antibodies is rare (≈10% of myasthenia gravis cases) (*7*), the potential role of HEV infection as a trigger of autoimmune disorders should be investigated. Some cases of anti-MuSK myasthenia gravis associated with HIV (*8*) or Epstein-Barr virus (*9*) infections have been reported. Nevertheless, our findings do not enable us to draw conclusions regarding causality. Our observation might suggest a coincidental temporal association between HEV infection and myasthenia gravis or a triggering of autoimmunity by HEV. Moreover, anti-MuSK myasthenia gravis is usually characterized by a rapidly progressive course with moderate to severe symptoms (*7*). The initial unusually benign clinical course in this patient might be explained by the effect of early ribavirin treatment or, more likely, by a particular type of MuSK antibodies (*10*).
